# Sweat as a diagnostic biofluid: analytical advances and future directions

**DOI:** 10.1016/j.jpha.2025.101473

**Published:** 2025-10-21

**Authors:** Dayanne Mozaner Bordin, Janice Irene McCauley, Eduardo Geraldo de Campos, David Bishop, Bruno Spinosa De Martinis

**Affiliations:** a Atomic Medicine Initiative, University of Technology Sydney, Ultimo, NSW, Australia; b Hyphenated Mass Spectrometry Laboratory, University of Technology Sydney, Ultimo, NSW, Australia; c Department of Forensic Science, Sam Houston State University, Huntsville, TX, United States; d Department of Chemistry and Fermentation Sciences, Appalachian State University, Boone, NC, United States; e Department of Chemistry, Faculty of Philosophy, Sciences and Letters of Ribeirao Preto, University of São Paulo, Ribeirao Preto, SP, Brazil

## Abstract

The use of sweat as an alternative specimen for biological analyses is well recognised and has been successfully applied in forensic toxicology and clinical health assessments. Compared to conventional matrices such as urine and blood, sweat samples are easy to obtain, are less invasive and present a reduced number of endogenous interferents. Advanced analytical techniques in sweat analysis have considerably expanded capabilities in the isolation of drugs, detection of complex metabolites and identification of biomarkers. The ability to detect a broader range of substances with greater sensitivity and accuracy, improved speed and efficiency and enhanced reliability present opportunities for non-invasive chemical sensing in sweat for a much broader scope of metabolites than traditionally recognised. The year 2023 was marked by an evolutionary step in artificial intelligence (AI), opening the door for improved pattern analysis and classification algorithms to improve diagnostic precision and therapeutic accuracy. It is anticipated that when combined with advances in the performance and miniaturization of integrated circuits, stretchable electronics, wireless connectivity and longer battery life, a substantial impact is expected on the development of wearable biosensor devices to provide meaningful information to the physiology of end-users, expanding its role in personalized medicine and health monitoring. Therefore, the aim of this review is to provide an integrative overview of sweat as a diagnostic and monitoring biofluid by first discussing traditional methods in clinical and forensic applications. Recent advancements in sweat biomarker detection are then highlighted, followed by an evaluation of sweat's potential for real-time physiological monitoring.

## Highlights

• Sweat is an accessible source of information for diagnostic & health monitoring.
• Advanced analytical techniques are expanding capabilities in biomarker discovery.
• A key challenge is the identification of biomarkers of clinical signification
• Improvements in AI are anticipated to enable advancements in diagnostic accuracy.

## Graphical abstract

**Figure:**
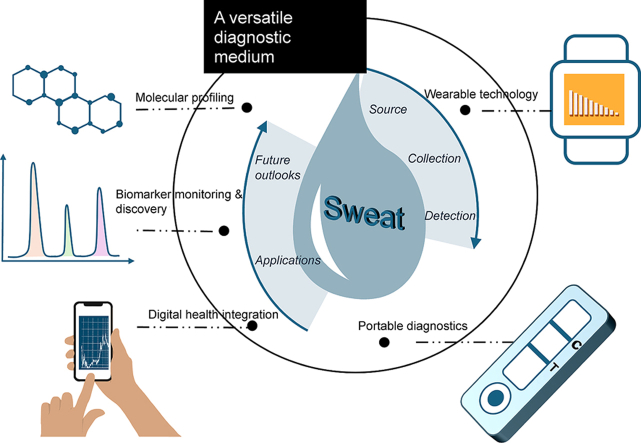

## 1 Introduction

The interest in alternative biological matrices such as sweat, oral fluid, breath and hair has long been recognised due to its sampling practicality (i.e., non-invasive, accessible, and low risk to the analyst), as well as its ability to provide health information. As a result, the integration of sweat sampling with wearable detection devices has been increasing throughout the past decade, gaining significant relevance. The recent article by Yang et al. [1] demonstrates the suitability of sweat as a detection medium for wearable sensors that interface with the skin and presents a promising and potentially inexpensive approach to diagnostics and health monitoring, particularly for large-scale studies related to public health or large populations. Analysing sweat also provides logistical benefits and can allow for efficient on-site procedures when collecting a large number of samples. As such, it is timely to explore the use sweat as a biofluid for analysis from an analytical perspective.

Sweat matrices present a range of advantages when compared to blood and urine, offering larger detection windows, diverse metabolic profiles and can contain a number of biomarkers that can provide information indicative of biological processes happening at a deeper molecular level [2]. For instance, sweat can represent a number of endogenous substances that are present in the blood, providing information on the physiological status for the individual, for example, electrolytes such as sodium (Na^+^) and potassium (K^+^). Daily water and electrolyte requirements are significantly affected by activity level, environmental conditions, and disease states and have been shown to be detectable in sweat during exercise using a wearable sensor (Table 1). Glucose, a key metabolite in routine health assessments, such as blood sugar testing for diabetes, can also be monitored in sweat. Zafar et al. [3] present a comprehensive review on wearable sweat-glucose sensors, but advancements continue to be made in this area (Table 1). Recently, a wearable electrochemical sensor integrated with iontophoresis was reported for non-invasive monitoring of sweat β-hydroxybutyrate (β-HB) which is used for diagnosis of diabetic ketoacidosis [4].

**Table 1:** Various metabolites detectable in sweat, including analytes, detection devices or mechanisms and reported analytical ranges and detection limits.

Analyte	Device/Mechanism	Detection limits and range (M)	LOD (M)	Refs.
β-hydroxybutyrate	Electrochemical senor	1 × 10^−5^ to 1.25 × 10^−4^	1 × 10^−5^	[4]
Inflammatory cytokines	SWEATSENSER (immunosensor)	0.2–200 ppt	ns	[5]
Opioids, stimulants: amphetamines; cannabinoids	Electrochemical sensor	ns	ns	[6]
Organophosphate, pyrethroid, and carbamate metabolites	GC-MS; LC-QTRAP	Variable reports 0.1–0.27 ppb	Variable reports 0.052–0.23 ppb	[[7], [8], [9]]
Na^+^ and K^+^ ions	SwEatch (electrochemical sensor)	1 × 10^−4^ to 1 × 10^−1^	ns	[[10], [11], [12]]
Glucose	Electrochemical non-enzymatic sensors	Dynamic range: 1 × 10^−5^ to 2 × 10^−3^	7.5 × 10^−7^	[13,14]
		Linear range: 5 × 10^−5^ to 6 × 10^−4^	3.2 × 10^−6^	[14]
	Colorimetric sensor	4 × 10^−5^ to 9 × 10^−4^	2.07 × 10^−5^	[15]

LOD: limit of detection; GC-MS: gas chromatography-mass spectrometry; LC-QTRAP: liquid chromatography-quadrupole-ion trap mass spectrometry; ppt: part per trillion; ppb: parts per billion; ns: not specified.

Wearable sensor devices are also being developed to monitor inflammatory cytokines, or biomarkers of inflammation. Variation in cytokine levels provide information on the diagnosis, stage and prognosis of various disease. For example Jagannath et al. [5] achieved an accuracy of more than 90% and specificity greater than 95% for a panel of cytokines including interleukin-6 (IL-6), interleukin-8 (IL-8), interleukin-10 (IL-10), and tumour necrosis factor-α (TNF-alpha) over an analytical range of 0.2–200 pg/mL with an immune sensor based on the interaction with specific antibodies. Sweat can also be used for the detection of drugs (e.g., amphetamines, opioids, and cocaine), as well as pesticides (Table 1). The review by Teymourian et al. [6] is comprehensive and presents the detectable levels for a number of illicit drugs in sweat. Sweat has also shown promise for the detection of pesticide exposure. Interestingly, the study by Genuis et al. [7] showed that sweat samples showed more frequently the presence of organochlorinated pesticides than serum or urine analysis. Thus, this biological matrix can enable the assessment of environmental and occupational exposures, clinical diagnosis, drug use and therapeutic monitoring within an analytical detection ranging from nanomolar up to millimolar concentrations (Table 1) [[4], [5], [6], [7], [8], [9], [10], [11], [12], [13], [14], [15]].

The composition of sweat, compared to other biofluids such as blood or urine, is less complex. It can be collected using absorbent pads or tubes. However, sweat analysis presents challenges such as low analyte concentrations and is susceptible to internal and external variables affecting sample collection. There is also a scarcity of studies providing complementary quantitative data making validation and standardisation difficult [16]. The dynamic nature of sweat composition further complicates reliable quantification due to evaporation, skin contamination, and handling challenges with small volumes. These factors also place additional demands on biosensors within wearable devices for accurately detecting sweat biomarkers [17]. Consequently, identifying clinically relevant biomarkers in sweat remains a significant challenge. Integrating sweat analyses with other traditional biological samples such as blood, urine, or oral fluid presents an opportunity to gather a larger and more informative data set, and with advancements in AI, could offer improved pattern analysis and classification algorithms [18]. Overcoming this challenge will further be supported by improvements that continue to occur in advanced scientific methods such as proteomics and metabolomics, which can deliver datasets that provide comprehensive information to understand complex biological functions and support biomarker discovery. This review aims to serve as a comprehensive resource for researchers interested in the interdisciplinary applications of sweat analysis, from an analytical perspective, covering the understanding of the matrix, how foreign substances (xenobiotics) enter and exit through sweat, as well as sampling, detection, and various applications.

## 2 Sweat physiology and xenobiotic transport

### 2.1 Glandular secretion

Sweat secretion is a homeostatic mechanism that plays a critical role in human thermoregulation, skin immunological protection and hydration. It is a clear biofluid secreted by the eccrine and apocrine glands. The mean pH is slightly acidic (pH range 4.0–6.8). Composed of 99% hypertonic aqueous solution, it also contains electrolytes (e.g., Na^+^, chloride (Cl^−^), and K^+^), urea, lactate, carbohydrates, proteins, peptides, amines, amino acids and, in smaller concentrations, inhibitors, antigens, antibodies, and other organic compounds such as excreted exogenous and exogenous substances [19].

The amount of sweat secreted is highly variable, depending on daily activities, emotional state, and environmental temperature. It is also dependent on other factors such as body location, hormonal imbalances, overactive thyroid gland and the sympathetic nervous system. The volume of sweat produced by an individual can be in the range of 0.2–3.0 L/h; however, during physical exercise, this volume can increase to > 3L/h [20].

**Fig. 1:**
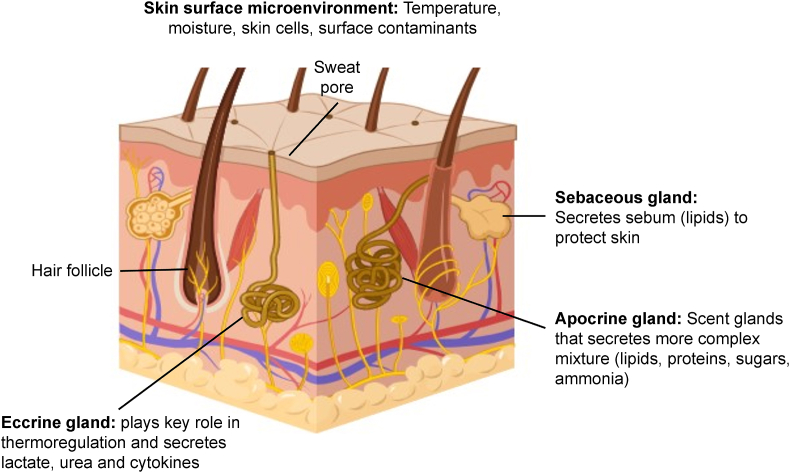
The human sweat gland anatomy and secretion pathways. The diagram illustrates the structure and distribution of eccrine, apocrine, and sebaceous glands in the skin, along with their respective secretory components [20,21]. Created in BioRender. McCauley, J. (2025) https://BioRender.com/pd86z2k.

The human body has 2–4 million sweat glands. These glands are classified into two types: eccrine and apocrine. The eccrine glands are the most numerous and are distributed throughout the body and are particularly abundant in the palms of the hands, soles of the feet, the face, and the chest. The apocrine glands are larger and located primarily in the axillae, pubis, and mammary glands. Both types of glands are derivatives of the epidermis located at different body sites. The secretory portion of the gland is in the dermis, and an excretory duct discharges the secretion at the surface of the skin that occur in almost every part of the skin. The development of sweat glands is in close association with hairs, and their ducts sometimes open into hair follicles (Fig. 1) [20,21].

Besides aqueous secretions, the skin also receives sebaceous secretions, especially on the face, scalp, and back regions. These secretions are sterile and odourless and are mainly composed of lipids. The major lipids are cholesterol (75%), triglycerides and fatty acids (20%). Blood capillaries nourish the sweat glands, in a similar way to the hair follicles [22]. The high lipid content of sebaceous secretions may transport and absorb many drugs and biomarkers. Sweat and sebum are mixed on the skin and are normally collected together. Most studies related to sweat sampling do not differentiate between both specimens and refer to this mixture as sweat [23].

### 2.2 Xenobiotic transport

The purpose of sweating is to maintain thermal homeostasis during physical or mental exertion or exposure to high temperatures; however, sweat also helps to excrete chemicals and metabolites such as excess micronutrients, metabolic waste and toxicants from the body. For example, a recent study by Brunmair et al. [24] correlated coffee consumption to the detection of caffeine components, specifically theobromine, theophylline, and paraxanthine with limit of detections (LODs) lower than 0.2 pg/μL in finger sweat. The research group further went on to develop a ‘Metabo-Tip’ assay for the analysis of fingertip sweat and found significant changes in the relative abundance of various metabolites in sweat composition following consumption of various foods and medications. They also found increased histamine in individuals with allergic reactions indicating the potential for sweat to support the development of predictive preventive personalized medicine [25].

The mechanisms of incorporation of substances, such as drugs, into sweat are not fully elucidated. Some of the main mechanisms include diffusion, active transport, receptor-mediated transport and metabolism. The enterohepatic system plays a role in the distribution, metabolism and elimination of substances from the body and can also influence the presence of certain substances in sweat. Enteric reabsorption occurs when a drug is secreted into the intestinal lumen and reabsorbed into the systemic circulation. The enterohepatic cycle is a key factor in the persistence of certain chemicals in the body, as it can slow the elimination of drugs, contributing to their long-term presence in tissues and potentially increasing the risk of side effects or toxicity. As a result, toxins may not be efficiently eliminated from the body and may accumulate in adipose tissue [26].

Substances can also accumulate in sweat by passive diffusion. Diffusion is the process by which a substance (e.g., drug) can passively move from an area of higher concentration to an area of lower concentration. If a drug is present in blood at elevated concentrations, it can diffuse through the walls of the sweat glands and into the sweat. In contrast, active transport is a process whereby certain drugs can be actively transported across the cell membrane of sweat glands, which involves energy consumption. This occurs through specific transporters, such as the P-glycoprotein (P-gp) transporter, which can pump drugs out of cells and into the sweat. Alternatively, some drugs can bind to specific receptors on the sweat gland cells and be transported across the cell membrane mediated by receptors. Interestingly, some drugs, such as amphetamines and opioids, can be metabolized by enzymes present in the sweat glands, which can result in the formation of new compounds that can be secreted into the sweat [27].

Not all xenobiotics are equally incorporated into sweat, and the amount may be influenced by physicochemical properties (e.g., molecular mass, pKa, protein binding and lipophilicity). Non-ionized, basic compounds and low protein binding drugs present a higher incorporation rate from blood to other fluids and/or cells. Therefore, smaller molecules that easily cross the membranes are expected to accumulate in sweat in greater concentrations than polar hydrophilic metabolites [28,29]. Some drugs, such as amphetamines, have been found to be present in sweat at concentrations that are similar to those found in blood. However, the interval between drug use and detection on the skin surface depends on the properties of the drug and on the sensitivity of the analytical method used. In chronic abusers, drug molecules are permanently present on the skin due to the stratum corneum acting as a temporary reservoir [23,30].

While sweat testing can offer a promising non-invasive method for drug detection, its effectiveness depends on the physicochemical properties of the drug. Factors such as lipophilicity, molecular size, and protein binding will influence how well a drug is incorporated into sweat, affecting its detection in different applications. However, some studies do appear to indicate that toxic elements and xenobiotics may be more readily excreted through human sweat. For example, a study by Genuis et al. [31] found that bisphenol A (BPA) was detectable in sweat even when absent in blood or urine, suggesting that sweat analysis may offer a more accurate reflection of BPA bioaccumulation. This was further supported by a related study by the same group reporting that several toxic elements, including heavy metals and metalloids, were preferentially excreted through sweat, sometimes appearing exclusively in this matrix [32]. This suggests that induced perspiration may be used as a means to eliminate bioaccumulated toxicants such as BPA and heavy metals. However, even as of 2020, our understanding of sweat composition and its diagnostic relevance remains incomplete. Beyond the well-characterized transport of Na^+^ and Cl^−^, which forms the basis of the widely accepted sweat test for cystic fibrosis (CF), the mechanisms governing the secretion and reabsorption of most other sweat solutes are still poorly understood. As a result, studies such as these highlight a need for furthering our understanding of the skin's excretory pathways, particularly regarding metabolites, pathogens, and xenobiotics.

## 3 Sweat as an alternative matrix: Advantages and disadvantages

Traditionally, in clinical and forensic analyses, whole blood and urine are matrices of choice with well-established applications. However, exploring alternative biological matrices to substitute or combine with blood and urine tests can help address known limitations, such as difficulties of field execution, complexity of the matrix or their overuse or reliance on as a basic screening tool for potentially serious pathologies.

Sweat testing has several advantages over blood and urine. First, sweat sampling is recognised to be minimally invasive, easy and does not require specialized personnel. As such, the collection does not need to be performed in a private space, which can be an advantage in some settings, such as workplace testing or in situations where privacy is a concern. Further, it contains less endogenous interferents, which facilitates easier sample preparation processes and therefore increases routine testing throughput. Manipulating sweat specimens presents a low risk of exposure to biological hazards (given that it is unexpected to contain pathogens) and is less susceptible to tampering (which is a known limitation of some biofluids, especially urine). In sweat, the primary components are typically the parent substances rather than their metabolites. This characteristic proves advantageous for new psychoactive substances (NPS), especially those with unknown metabolic pathways. Sweat testing offers a longer detection window compared to urine or blood tests [33].

Although many advantages make sweat an excellent biological matrix, its use has some inherent limitations. The disadvantages include a lack of information about dose-response relationships, thus the concentrations of drugs in sweat cannot be used to estimate intake (dose) or impairment. In general, the concentrations of some drugs of abuse in sweat are in the ng/mL range, requiring sensitive analytical methods. Comparatively, the volume of whole blood and urine specimens collected for analysis is, in general, enough to perform more than just one single analysis. However, with sweat, if just a single patch is collected, it is not possible to perform a second extraction, unless the extraction is performed with just a portion of the patch (e.g., half of the patch) [33] or if more than one patch is collected from the individual. Sweat analysis can also be prone to environmental contamination if not handled correctly. Furthermore, access to laboratories capable of performing this analysis is essential, and the limited number of facilities offering sweat testing may hinder its implementation and routine application.

The volume of sweat can also be affected by different factors, including interindividual variation, physical exercise condition and environment temperature, and subsequently, require consideration when determining how the sweat should be collected. For example, Nunes et al. [34] developed a screening method to detect 26 target molecules in a single analytical run using liquid chromatography-tandem mass spectrometry (LC-MS/MS). Two sampling techniques were also compared (patches versus direct sampling) from either the chest or back. The study found that an advantage of patch-based sampling is that it can be used under both rest and exercise conditions; however, a disadvantage is that it may introduce interferences. Conversely, the advantage of vial-based direct sampling is that it avoids such interferences, but a disadvantage is that it only yields sufficient sweat for analysis after exercise [34].

Yielding enough sweat, regardless of the situation, is a very typical problem and remains a key challenge. For example, the pilocarpine iontophoresis sweat test is widely regarded as the gold standard for diagnosing CF. However, its effectiveness is limited by the requirement for specialized equipment and the difficulty in obtaining sufficient sweat from infants and young children [35]. Therefore, a recent study by Chen et al. [35] developed a skin patch with dissolvable microneedles (MNs) containing pilocarpine that eliminates the equipment and complexity of iontophoresis. Upon pressing the patch to skin, the MNs dissolve in skin to release pilocarpine for sweat induction. Results showed that the amount of pilocarpine delivered and sweat output for the two methods was comparable. The patches were also shown to be easy to use with good tolerability in subjects (i.e., little to no pain and only mild transient erythema). However, the sweat Cl^−^ concentration was subsequently highly for the patch with the MNs with the exact cause yet to be defined and warranting further investigation [35].

Considering the variability in individual sweat composition, which can be influenced by factors such as sweat flow rate, skin surface contamination, sebum secretion, and metabolic byproducts, as well as methodological variations in sweat collection and/or analysis, these variables must be carefully addressed in any analytical workflow.

## 4 Sweat sampling and analysis

### 4.1 Sweat sampling

Sweat sampling techniques have undergone significant advancements in recent years, offering various methods for the collection of sweat samples from the human skin. It is noteworthy that the collected sample consists of a mixture of sebum and two different types of sweat, which vary based on the location of the skin due to uneven distribution of sweat glands throughout the body. To facilitate sweat collection, a wide range of devices have been developed (Table 2) [[36], [37], [38], [39], [40], [41]].

**Table 2:** Commercially available sweat collection devices.

Sweat collection device	Collector	Volume (μL)	Collection period (h)	Detection mechanism	Refs.
Macroduct®	Elliptical shaped with capillary plastic coil	60	0.5	Sweat stimulation with pilocarpine; sweat transferred to micro-centrifuge tube for analysis.	[36,37]
PharmChek®	Adhesive dermal patch with absorbent pad	70–80	168–240	Immunoassay for screening (different panels available and LC-MS/MS∗ for confirmation in certified labs)	[38,39]
DrugWipe®	Absorbent pad with lateral flow indicator	10–15	Variable#	Immunochromatographic and colorimetric	[40]
Gx Sweat Patch®	Adhesive patch with integrated absorbent pad	50–60	<336	Colorimetric assay; mobile app	[41]

LC-MS/MS: liquid chromatography-tandem mass dpectrometry; # depends on sweating.

Direct collection is one of the early methods adopted for collecting sweat samples. The collection is performed using the passage of gauze or swabs over the skin, followed by redissolution of the sample in water or, pipetting sweat followed by transfer to glass tubes [37]. The analysis of sweat specimens collected through direct swab collection can provide information about drug exposure with the advantage of being minimally invasive. However, there are some caveats. It is recommended to control the use of topical products, pharmaceutical drugs and diet and, to detail the axillary hair shaving process [37].

**Fig. 2:**
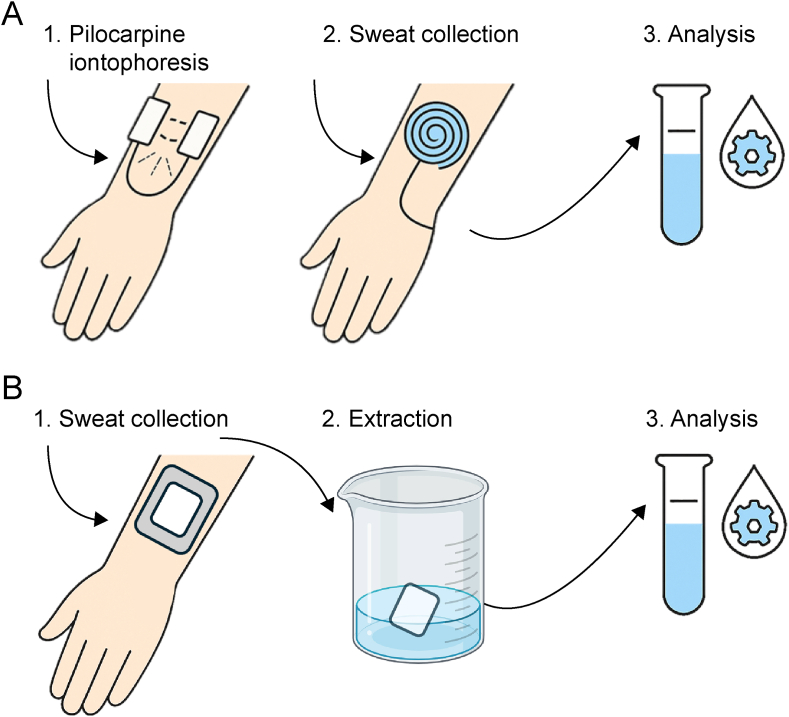
Direct sweat collection devices. (A) Schematic representation of the Macroduct® (ELITechGroup Inc.) sweat collection system (1), capillary tube collection (2), and direct analysis of the sample (3) [36]. (B) Schematic representation of the PharmChek® (PharmChem Inc.) sweat patch, which passively collects sweat on an absorbent pad during wear (1); after removal, the absorbent pad is excised and extracted (2), followed by laboratory analysis for target substances (3) [38,39]. Created in BioRender. McCauley, J. (2025) https://BioRender.com/pd86z2k.

The Macroduct® (ELITechGroup Inc.) is a sweat collection system, which comprises sweat stimulation and collection. It was originally designed for CF diagnostics, but it has also been applied for metabolomic studies. The sweat collection device is composed of a 29 mm diameter disk equipped in its interior with a capillary plastic coil that collects sweat [36] (Fig. 2).

Pilocarpine administration through the skin by electric current (iontophoresis) is responsible for the sweating induction. This substance is a muscarinic agonist, which rapidly stimulates the localized production of sweat. Generally, it is placed in the forearm of the individual and 60 μL of fluid can be collected in 30 min. Since iontophoresis is based on sending electrical current to the skin, the process may be uncomfortable for some individuals. Otherwise, sweat can be readily collected, and standard sample volume can be applied for following analysis [[36], [37], [38], [39], [40], [41]].

Sweat patches are adhesive dermal constructs designed to facilitate the collection of sweat. PharmChek® (PharmChem Inc) patches are another example of a sweat collection device that are commercially available [38,39]. The sweat patch consists of an adhesive layer on a thin transparent film of polyurethane waterproof surgical dressing to which a rectangular absorbent pad is attached (Fig. 2) [[38], [39]]. The analytes are deposited into the absorbent bandage of cellulose that is adhered to the skin by an adjoining adhesive. A polyurethane layer protects the device from external contamination, while allowing the gas exchange between skin and environment, not harming the skin [42,43]. The sweat patch acts as a specimen container for non-volatile, liquid components of sweat, including doping agents. Non-volatile substances from the environment cannot penetrate the transparent film, which is a semi-permeable membrane over the pad that allows oxygen, water, and carbon dioxide to pass through the patch, leaving the skin underneath healthy. Over a period of several days, sweat saturates the pad and drugs present in sweat are accumulated and retained. Prior to the application of the adhesive, the skin is cleaned with isopropyl alcohol to remove existing contaminants. The PharmChek® patches allow one to carry out normal activities including swimming and showering without the need for removing the patch. Since the device can stay attached to the skin for up to 14 days, monitoring can be performed for a longer period. It also presents some features that inhibit sample adulteration. For example, once removed the sweat patch cannot be attached again onto the skin and each device has a unique serial number printed, ensuring the chain of custody. A wide-range detection panels are available to detect common classes of drugs.

**Fig. 3:**
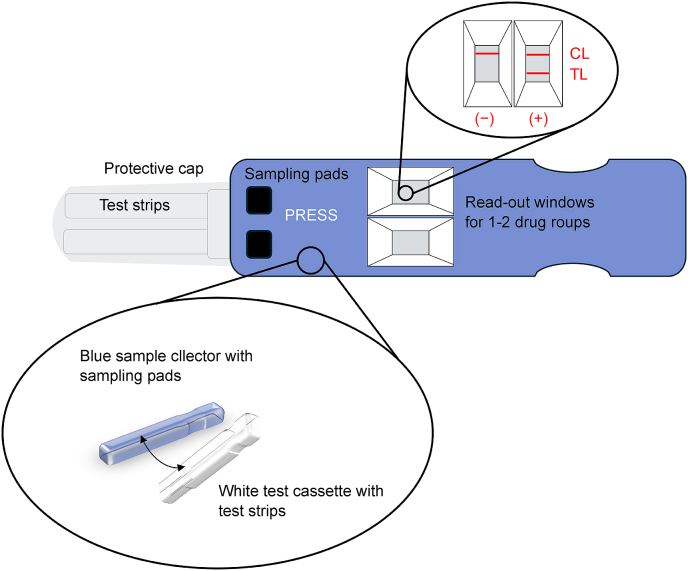
Schematic representation of the DrugWipe® A (Securetec Detektions-Systeme AG) immunological screening test. The blue sample collector is first removed from the test cassette. For skin or surface testing, the sampling pads are moistened with water; for saliva, the dry collector is used directly. After sample collection, the collector is reinserted into the cassette and the test strip is immersed in water for 15 s. Results are visually interpreted after 5 min based on the appearance of test lines. Adapted with permission from Ref. [40].

DrugWipe® (Securetec Detektions-Systeme AG) is a compact device resembling a pen, designed for non-invasive, on-site testing, applicable to sweat testing (Fig. 3) [40].

It is based on immunochromatographic testing, which allows for the immediate detection of different classes of drugs (opiates, amphetamines/methamphetamines, benzodiazepines, ketamine/phencyclidine (PCP), buprenorphine, and methadone) both on body surfaces and in solution. Similarly, to several other lateral flow immunoassays, the device utilizes colorimetric changes to identify test results, in which a transition from pink to red indicates the presence of drugs, while the absence of colour indicates the absence of drugs in the collected sample. The collection and analysis are straightforward and rapid, with results typically obtained within 3 min [[44], [45], [46], [47]].

**Fig. 4:**
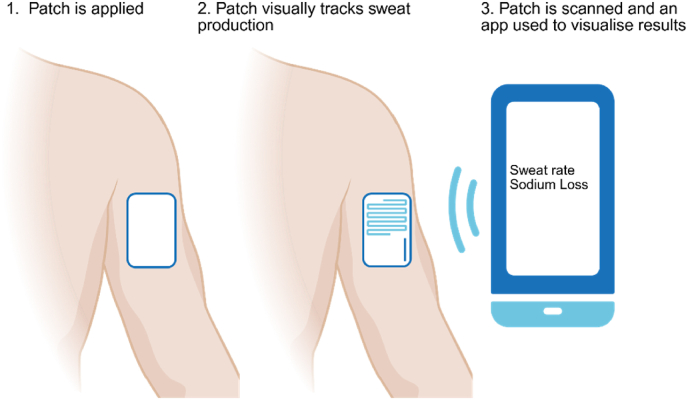
Schematic representation of the Gatorade Patch-On Sweat Collection System (Gx Sweat Patch) (Gatorade) for sweat monitoring. (1) The patch is applied to the skin prior to physical activity, where it absorbs and undergoes a colour change that visually reflects sweat production. (2) After exercise, the patch records sweat composition, including fluid and sodium loss, in a format that be digitally interpreted. (3) A personal device scans the patch to provide quantitative results, such as sweat rate and electrolyte loss [41]. Created in BioRender. McCauley, J. (2025) https://BioRender.com/pd86z2k.

In specific instances, sweat patches also incorporate absorbent materials and channels or microfluidic systems to enhance the transport of sweat towards the reservoir for collection. An example of commercially available patches is the Patch-On Sweat Collection System (Gatorade, Chicago, IL, USA) [41]. The Gatorade Patch-On Sweat Collection System (Gx Sweat Patch) is a wearable device designed for sweat sampling during physical activity. It consists of a flexible patch integrated with microfluidic channels and absorbent pads. The microfluidic channels facilitate the flow of sweat into the absorbent pads, ensuring reliable and controlled sample collection (Fig. 4) [41].

The system is easy to use and can be applied to various areas of the body. A mobile app delivers real-time information and lets users create personalized hydration and dietary plans. It works by measuring sweat rate and Na^+^ loss during exercise. After a workout, the patch's colorimetric reaction is scanned using the Gatorade Gx mobile app, which interprets the data and provides feedback to the user [41]. Gatorade has demonstrated the effectiveness of this system in monitoring hydration status and electrolyte balance in athletes [48].

Apart from the commercially available sweat devices, the research into wearable devices continues to progress and improve detection of a wide range of analytes, including electrolytes (Na^+^, K^+^, Cl^−^), endogenous metabolites (e.g., glucose, lactate), and biomarkers relevant to medical conditions (e.g., cortisol, pH levels) [49]. They have shown promise in applications such as sports performance monitoring, disease diagnosis and personalized healthcare management [50]. Advanced systems often integrate sweat sampling and analysis using technologies like microfluidics, biosensors, or lab-on-a-chip platforms [51].

### 4.2 Sample preparation

Sweat analysis quality relies on the efficiency of sample collection and preparation and subsequent selectivity and sensitivity of the analytical methods. Although direct sweat analysis is common, achieving successful analyte detection and minimizing matrix interferences necessitates meticulous sample processing. This ensures that detection levels are met and unwanted compounds in the matrix are effectively removed. Various techniques are employed for sweat clean-up and analyte enhancement detection.

Liquid-liquid extraction (LLE), stands out as a well-established and widely used technique. It involves the use of analyte-specific solvents, including aqueous phosphate buffers and organic solvents such as methanol and acetonitrile [52]. In certain instances, the discriminatory capacity of detectors for drugs, xenobiotics, and electrolytes is enhanced through derivatization during sample preparation, especially for GC. This additional step allows for improved detection and/or separation, contributing to the overall sensitivity and specificity of the analytical methods employed. This approach has proven effective in extracting a diverse array of compounds from sweat samples. For example, Delgado-Povedano et al. [53] compared different sample preparation strategies to maximise the detection of metabolites, with a particular emphasis on non-polar compounds in sweat from humans after exercising. The study used solvents with differing polarities and found methyoxiamination plus silylation derivatization after LLE with dichloromethane was the best option to obtain a representative snapshot of sweat metabolome collected from different body parts after moderate exercise [53].

Another prevalent technique is solid-phase extraction (SPE), which utilizes cartridges consisting of silica base and/or copolymer solid phases. This method ensures efficient extraction and purification of target analytes, contributing to the overall robustness of the analytical process [33]. The use of copolymer-containing cartridges enhances specificity in isolating compounds of interest, thereby facilitating subsequent analysis. SPE is particularly useful for selective extractions where target analytes are typically present at low concentrations. SPE enables preconcentration prior to detection and is especially advantageous when working with complex matrices that may contain interfering compounds or when analytes are present at trace levels. DPX, also known as dispersive pipette tip SPE, is a new miniaturized SPE extraction system. It involves a pipette tip packed with sorbent material between two filters, allowing for rapid and efficient mixing of the sample and sorbent through air aspiration. This process enhances analyte interaction and enables quick extraction, even from low-volume or complex matrices [54]. A recent study by Gomes et al. [55] using disposable pipette extraction (DPX) for extraction and concurrent analysis of fourteen psychoactive substances in the sweat of drug users. The study by Gomes et al. [55] achieved quantification limits between 2 and 30 ng/patch, with linearity from 2 to 1100 ng/patch. The method was then successfully applied to 30 sweat samples from drug-using volunteers to successfully detect and quantify multiple analytes with recovery rates from 72.4% to 97.1%. The optimisation of this approach was then reported for the extraction of basic and neutral psychoactive substances using gas chromatography-mass spectrometry (GC-MS) and design of experiment approach (DoE) [56].

### 4.3 Normalization

However, the variability that occurs in both sweat composition and volume inherently affects the reliability of quantified analytical outcomes. Normalization of sweat volume is required to achieve authentic and comparable results. Appenzeller et al. [57] evaluated whether naturally occurring Na^+^ and K^+^ in sweat could be used as internal references to estimate the volume of sweat collected on a patch. They found that Na^+^ can serve as a reference point to estimate the volume of sweat collected. When factors such as gender, skin region, and sweat collection site are considered, Na^+^ concentrations show low inter-individual variability. As a result, this approach may enable more accurate quantification of xenobiotics in sweat samples. This method involves determining Na^+^ and K^+^ levels through capillary zone electrophoresis coupled with a diode array detector (CE-DAD) set at 214 nm for the purpose of normalizing sweat volume [57]. Although the use of Na^+^ concentration for normalization is proposed as a preferable option over K^−^ concentration, it is important to highlight that the normalization of the sampled sweat volume remains an evolving field of investigation. It is noteworthy that the application of normalized sweat volume for CF diagnosis has not been implemented to date, highlighting the evolving nature of this concept [58].

### 4.4 Single-detection systems

The complexity of sweat analysis instrumentation varies depending on the target analytes. Commercially available specialized single-detection systems utilize a variety of measurement principles, including potentiometry (e.g., Na^+^), amperometry (e.g., glucose), colorimetry (e.g., lactate) conductivity (e.g., Cl^−^), differential pulse voltammetry (e.g., cortisol), or osmolarity (e.g., total concentration). These techniques may be employed individually or in combination to achieve comprehensive analysis of the targeted analytes. These detections are commonly utilised in wearable sensors by incorporation of ion-selective electrodes or conductivity electrodes, enzymatic sensors or visual colorimetric reactions (e.g. pH change). These sensors can be incorporated in wearable sensors or devices for real-time and continuous monitoring of sweat composition.

### 4.5 Advanced analytical techniques

Advanced analytical techniques also play an important role in sweat diagnostics and biofluid monitoring. While not suitable for portable devices, these techniques contribute to more comprehensive sweat analysis and enable biomarker identification, which can then inform the development of future sensor technologies. The use of advanced analytical techniques in sweat analysis has considerably expanded capabilities in the isolation of drugs, detection of complex metabolites, and identification of biomarkers. These techniques, while integral to doping control, also play a an important improving our understanding of the pathophysiological mechanisms underlying a variety of disorders [59]. Nuclear magnetic resonance spectroscopy (NMR) and MS, coupled with separation techniques (GC, LC and CE), are the main techniques used. Despite its lower sensitivity, NMR spectroscopy provides a swift, reproducible, and non-destructive platform for exploring the sweat metabolome. For example, high-resolution ^1^H NMR spectroscopy combined with statistical pattern recognition has been used to generate sweat spectra and quantify major metabolite classes, including lactate, amino acids, and lipids [61]. The study observed similar concentration changes in analytes using both NMR analysis and statistical methods such as principal components analysis (PCA), discriminant analysis of principal components (DAPC), and partial least squares-discriminant analysis (PLS-DA). These approaches were applied to sweat samples from healthy male and female participants, revealing no significant differences in metabolite composition between sexes. However, a high degree of interindividual variability was observed, which did not correlate with clinical information and was speculated to relate to factors such as hydration status and dietary intake [61]. Meanwhile, MS is known for its versatility and can be used to provide structural information and, with the availability of spectral libraries, can assist with compound identification in sweat samples.

The gold standard for analysing volatile organic compounds (VOCs) in sweat for assessing cancer biomarkers and discerning sex-related differences in the human sweat volatilome remains to be GC-MS [[60], [61], [62]]. LC, with recent MS advancements including highly sensitive triple quadrupoles (QQQ) and high-resolution mass spectrometers (HRMS) such as ion-trap, and time-of-flight instruments (TOF), has significantly improved the coverage and specificity in sweat metabolome and proteome analysis. One of the most selective separation techniques, CE-MS is another approach that can provide valuable information into the sweat metabolome, particularly for polar metabolites. Further, to aid protein separation and identification for downstream mass spectrometric analysis, two techniques are commonly used: two-dimensional gel electrophoresis (2-DE) and liquid chromatography, especially reversed-phase liquid chromatography (RP-LC) [63,64]. HRMS, whether through "top-down" or "bottom-up" approaches, assists in protein identification and quantification. Tandem mass spectrometry (MS/MS) spectra acquisition is achieved through data-independent acquisition (DIA), data-dependent acquisition (DDA), and multiple reaction monitoring (MRM), offering detailed information into eccrine sweat protein differentials. Advancements in high-resolution detection and separation techniques have significantly enhanced the ability to identify biomarkers and complex metabolites in sweat. This knowledge is an essential first step for guiding the development of wearable sensors for a more diverse array of biomarkers into compact, wearable devices for application.

#### 4.5.1 Sweat metabolomics

Metabolomics has emerged as a complementary tool to genomics and proteomics and can provide information to further understand pathological conditions and individual specific physiological responses. This field has also expanded to include biometric identification research. In contrast to plasma or urine, sweat presents with a less complex matrix, making it conducive for biomarker discovery. The dynamic nature of sweating in various pathological conditions positions sweat as a valuable resource for comprehending pathophysiological alterations through metabolomics. Despite its potential, challenges in sweat metabolic profiling include understanding variations across individuals and collection locations, comprehending physiological concentration fluctuations, addressing the low collection volume, and considering the impact of diseases [65].

For example Kutyshenko et al. [66] conducted a seminal study on sweat metabolomics using NMR. Through the collection of samples from diverse body locations, they identified major constituents such as lactate, glycerol, pyruvate, and serine. Notably, variation in the individual signal intensities was observed indicating that sweat metabolic profiles are relatively conserved in various parts of the human body with the exception the foot region, which are characterized by a unique metabolic pattern.

In order to study individual repeatability and what elements of an individual signature remains constant, Xu et al. [67] utilised an alternative method that utilizes dissimilarities between samples in order to overcome sampling issues and susceptibility to numerous influencing factors. They performed a large-scale study of human sweat of nearly 1000 GC-MS analyses from 200 individuals, sampling once per fortnight over a 10-week period. The application of similarity-based approaches (PCA and the Kolmorogov-Smirnov rank test) to the GC-MS of sweat profiles suggested that characteristic individual signatures could be deduced indicating the applicability of this approach to further elucidate the potential effects of genetics, environment and other personal factors on sweat composition if conducted on a much larger study [67]. Further, in a metabolomics study employing chemical isotope labelling (CIL) with liquid chromatography-quadrupole time-of-flight mass spectrometry (LC-QTOF-MS), Hooton et al. [68] detected a myriad of metabolites through the application of a gauze patch for sample collection. Subsequently, Hooton and Li [69] extended this approach to provide metabolic profiles for different skin locations, adding to our understanding of localized variations in sweat composition. Hair et al. [70] and Greco et al. [71] have also demonstrated the feasibility of sweat as a biometric marker, specifically utilizing metabolite levels of lactate, urea, glutamate, and L-alanine. Employing colorimetric reactions, ultraviolet spectrophotometry, and GC-MS the authors demonstrated that the combination of different levels of these metabolites established a reliable and distinctive profile to differentiate each participant.

Attention has also been directed towards identifying biomarkers associated with cancer and skin conditions such as atopic dermatitis. Calderón-Santiago et al. [72] investigated lung cancer biomarkers through the analysis of sweat chemical profiles. Using LC-QTOF-MS, specific metabolites such as trihexose, a tetrahexose, suberic acid, MG (22:2), and nonanedioic acid were identified as potential diagnostic markers. Regarding skin conditions, the study conducted by Agrawal et al. [73] showed that the lipid profile in sweat was characterized for individuals with and without atopic dermatitis, with a focus on oxygenated lipids, endocannabinoids, and ceramides/sphingoid bases. Utilizing LC-MS/MS, the researchers profiled over 100 lipid mediators, detecting 58 in sweat. Elevated concentrations of C30–C40 ceramides, ceramides in general, and C18:1 sphingosine were observed in the sweat of participants with atopic dermatitis, particularly in men. The findings suggest that sweat lipid profiling could serve as a non-invasive diagnostic for the early detection and implementation of intervention strategies for atopic dermatitis.

The integration of metabolomics into sweat analysis demonstrates its potential as a non-invasive tool for personalized biochemical insights and contributing to the development of innovative diagnostic strategies. Its application potential range widely, including strengthening security measures in controlled environments, as well as providing support for legal investigations.

#### 4.5.2 Sweat proteomics

Over the past decade, significant progress in omics high-throughput technologies have significantly enhanced the characterization and analysis of biological components, including the sweat proteome [74]. This advancement extends to an in-depth analysis of sweat biomolecules, offering an accelerated pathway to further understand the pathophysiological processes associated with various disorders and facilitating biomarker identification [75]. Additionally, omics methodologies contribute to a comprehensive understanding of the crosstalk between the skin microbiome-body relationship and the alterations in the proteome-metabolome under diverse physiological conditions [76]. Despite the numerous studies investigating the applications of omics techniques in systems biology and biomarker discovery, with a primary focus on protein-metabolic alterations in common biofluids such as plasma and urine, sweat has received comparatively less emphasis in existing literature [[77], [78], [79]].

Recent research findings indicate substantial variations in the protein composition of human sweat in various disorders such as schizophrenia, atopic dermatitis, CF, tuberculosis and immune diseases such as Vogt–Koyanagi–Harada (VKH). Raiszadeh et al. [80] pioneered a large-scale investigation into the sweat proteome using LC-MS/MS proteomics analyses on endocrine sweat from healthy volunteers and individuals diagnosed with schizophrenia. Global proteomic analysis revealed distinct differences between proteins in sweat and serum. Moreover, the comparison between populations identified 17 proteins present approximately twice as much in the sweat of individuals with schizophrenia compared to those without, supporting the use of sweat as a promising matrix for identifying proteins influenced by disease-related biochemistry. In a separate study, Ono et al. [81] explored the chemical composition of sweat in volunteers with atopic dermatitis using NMR. The investigation uncovered elevated glucose levels and implicated the glucose transporter GLUT2 in the underlying mechanism.

Burat et al. [82] employed shotgun proteomics to analyse sweat from 28 healthy volunteers and found 987 unique proteins and 344 common proteins, with connections to physiological processes, such as oxidative stress. In a subsequent study, Burat et al. [83] used advanced proteomics to investigate sweat samples from both healthy individuals and CF patients. The study identified distinct proteins between non-CF and CF patients, including variations between CF genotypes and tentatively identified nine potential CF diagnosis biomarkers and seven potential CF severity biomarkers, warranting further exploration [83].

Adewole et al. [84] explored eccrine sweat as a potential source for diagnostic biomarkers of active tuberculosis (TB), given the common symptom of excessive sweating in TB patients. A global proteomic analysis of eccrine sweat was conducted from individuals with active pulmonary TB, non-TB lung diseases, and healthy controls. The comparison of proteomic profiles identified over 100 unique proteins in the sweat of all three groups, with 26 proteins exclusively detected in active TB patients. These unique proteins were associated with immune response, auxiliary protein transport, and cellular components like ribosomes. The findings support the potential of sweat proteomics for identifying specific biomarkers of active TB and the potential for the development of a non-sputum based diagnostic test [84].

In an investigation into VKH disease, an autoimmune condition associated with visual impairment, Cui et al. [85] conducted a proteomics and metabolomics analysis on sweat samples from both VKH patients and normal controls. Using LC-MS, 716 proteins and 175 metabolites were identified. Significant differences were observed in 116 proteins (99 decreased, 17 increased) and 21 metabolites, including reduced levels of choline, L-tryptophan, betaine, and L-serine in VKH patients. The findings suggest an important role for the amino acid metabolic pathway in the pathogenesis of VKH disease [85].

Whilst biofluids like plasma and urine are typically the more preferred sample type for biomarker investigations, as can be seen, the recent literature presents significant variations in the protein composition of human sweat across a diverse number of disorders, supporting sweat biofluid as a suitable matrix for identifying disease-related biomarkers and future potential for a non-invasive diagnostic tool.

## 5 Sweat analysis applications

Sweat analysis has gained traction across a range of clinical and diagnostic applications and include established methods to monitor electrolyte levels in CF, glucose and metabolite profiling in diabetes, as well as drug detection in forensic toxicology. Over the past few decades, research has expanded to include neurodegenerative conditions such as Alzheimer's (AD) and Parkinson's disease (PD). Other areas of investigation are also emerging such as the profiling of VOCs and trace metals.

### 5.1 Forensic applications

The use of sweat in toxicological applications is still not widespread. However, sweat appears as an object of great interest in this area especially when considering the analytical advantages such as the information potential regarding the cumulative register of exposure to substances and the simple sample collection. Drug screening in sweat samples can be especially useful in criminal justice settings, for example when the probation office needs to evaluate drug use by individuals on parole. Further, drug use monitoring is commonly needed in rehabilitation treatment programs and workplace drug testing. Sweat testing can also be used to evaluate a worker's exposure to substances in the workplace. In doping control, the standard biological matrix to be tested is urine. Urine testing provides information on recent drug use (1–7 days) depending on the type of drug used and the frequency, thus making test scheduling an issue for many applications. In addition, this matrix is susceptible to tampering, which can result in major issues for doping control organizations [86,87]. In such situations, sweat could be employed in the monitoring of the intake of doping agents with reduced possibility of patch sample tampering when compared to urine [88].

It has been demonstrated that sweat can be used for qualitative and quantitative analyses of psychoactive substances. Delta-9-tetrahydrocannabinol (THC) has been found in sweat specimens (directly collected by swabbing the forehead with a cosmetic pad) collected from injured drivers in France. Concentrations ranged from 4 to 152 ng/pad, whereas 11-nor-9-carboxy-THC (COOH-THC) and 11-hydroxy-THC (OH-THC) were not detected [89]. A disadvantage identified in this paper is that the volume of sweat is highly variable and the volume of sweat collected within the pad is unknown and results should be considered as qualitative or semi-quantitative [89]. Moreover, the concentration of THC in sweat can be rather low and, is another limitation, especially if direct analysis (immunoassays) is performed. For example, in a study with volunteers who reported recent drug use, although THC had been detected in oral fluid, subsequent tests using DrugWipe® were negative [47].

Stimulants have also been detected in sweat specimens collected using PharmCheck® patches from athletes by GC-MS. Cocaine (150–300 ng/patch), amphetamine (100–300 ng/patch), 3,4-methylenedioxymethamphetamine (MDMA) (115–200 ng/patch), methylphenidate (135 ng/patch) and mephentermine (150 ng/patch) were quantified in the specimens [88], suggesting that basic, parent drugs can be detected in authentic sweat specimens collected using patches. Similarly, two volunteers were administered 100 mg of MDMA and sweat testing conducted in the armpit region of the body using DrugWipe®, with positive results being obtained as early as 2h after administration and up to 12h [45]. Interindividual variations can play a role in final concentrations and detection windows in sweat. In synthetic sweat, cocaine, and its metabolite benzoylecgonine, amphetamine, methamphetamine, 3,4-methylenedioxyamphetamine (MDA) and MDMA were detected by GC-MS with limits of detection between 3.7 and 14.3 ng/pad, using the DrugWipe® 5A device [90]. The limits of detection for COOH-THC and morphine were 4.2 and 12 ng/pad [90].

Cocaine and its metabolites, and opioids have been detected in sweat specimens collected from opioid-users and pregnant women who were under treatment with buprenorphine via LC-MS/MS [91]. The concentration ranges varied depending on the analyte. For example, cocaine was detected in 15 samples with concentrations between 1 and 1420 ng/patch whereas the concentration of buprenorphine ranged from 1 to 2.3 ng/patch (*n* = 12) [91]. Using a GC-MS-based method, a set of opioids and cocaine and its metabolites were detected in sweat patches, reaching limits of detection as low as 1.25 ng/patch for codeine and morphine; 2.5 ng/patch for cocaine, benzoylecgonine, ecgonine methyl ester, anhydroecgonine methyl ester, 6-acetylmorphine and heroin; and 5 ng/patch for methadone and 6-acetylcodeine [92]. The authors state that sweat testing overcomes a known limitation when urine and blood are tested for opioids. For example, heroin and 6-acetylmorphine can be detected in sweat (as demonstrated with this method), which is usually not possible in blood or urine. This can be helpful to differentiate illicit use of heroin from other situations where morphine can be detected in a biological sample (e.g., poppy seeds consumption) [92].

To address the issue of low concentration of drugs in sweat samples, the study by Gomes et al. [56] developed and optimized two GC-MS methods using DPX sample preparation to extract basic and neutral psychoactive substances from sweat sample, resulting in increased analyte signals by 54.7% for basic compounds and 39.2% for neutral compounds. This results in a direct improvement in method performance parameters, as stronger signals make it easier to detect low concentrations of analyte, improve signal-to-noise ratios, and enhance accuracy and confidence in quantification. Additionally, higher signal intensities tend to produce more consistent data, improving reproducibility and ultimately lowering the detection limit. As a result, the researchers were able to identify various psychoactive substances, including both traditional drugs and NPS such as dibutylone, *N*-ethylpentylone, 25E-NBOMe, 25C-NBOMe, 2C-C, and 2C-E. This is a significant achievement, as many NPS are present at low concentrations, even in blood, and monitoring their use through sweat analysis can provide complementary information on recent use alongside blood or urine testing [55,56].

For estimation of blood alcohol, Phillips and McAloon [93] tested alcohol consumption by applying a sweat patch fixed for one week on the individual, followed by chromatography analysis. The author suggested that the developed device may not be adequate for an accurate estimation of the consumed dose of alcohol [94]. In this case, the reabsorption of ethanol from the sweat patch (back diffusion) could be the cause of these findings [95]. Further, Kamei et al. [96] designed an equipment capable of continuous quantitative detection of ethanol in perspiration. Their study specifically demonstrated a correlation between ethanol sweat levels and those found in blood samples, presenting a compelling avenue for non-invasive monitoring of alcohol consumption. This finding laid the groundwork for subsequent investigations into the potential of sweat as a reliable matrix for assessing various substances.

Building upon this foundation, recent studies have employed diverse methodologies to enhance the scope and precision of toxicological investigations utilizing sweat. Schummer et al. [97] explored the analysis of ethyl glucuronide, a specific metabolite of ethanol, in sweat collected with PharmChek® patches. Their approach involved washing the patch with water, followed by SPE and analysis using GC-MS. This methodology allowed for a more detailed understanding of ethanol metabolites in sweat, contributing to the evolving landscape of sweat-based toxicological assessments. Gamella et al. [98] took a distinct approach by developing a biosensor for monitoring alcohol in sweat. Their biosensor, based on a bioenzyme composite graphite-Teflon electrode, Ag/AgCl reference electrode, and Pt electrode as an auxiliary, exhibited high sensitivity. The sweat stimulation was achieved through pilocarpine administration and collection using the Macroduct® 3700-SYS system. This innovative biosensor not only highlighted the potential for real-time monitoring of alcohol levels in sweat but, also the adaptability of biosensing technologies in the field of sweat-based toxicological assessments. Selvam et al. [99] contributed to the field by creating a wearable sensor specifically designed for ethyl glucuronide measurement in sweat. The device utilised two co-planar sensors modified with gold and zinc oxides, enabling the detection of ethyl glucuronide through a chemo resistive sensing process. This wearable sensor used monoclonal antibodies sensitive to the metabolite, identifying an innovative approach to facilitate convenient and continuous monitoring of specific substances in sweat.

### 5.2 CF

CF, a hereditary condition prevalent among individuals of Caucasian descent, stands as the most frequently occurring life-shortening autosomal recessive disorder [100,101]. It results from a mutation in chromosome 7 at position 7q31, affecting the transcription of the CF transmembrane conductance regulator (CFTR), a transmembrane protein essential in ion transport. The diagnosis of CF presents challenges due to the extensive phenotypic variations observed [101]. Currently, over 2100 mutations in the CFTR gene have been identified, emphasizing the genetic complexity of the disorder [102].

The malfunction of CFTR leads to reduced Cl^−^ excretion into the extracellular space, causing an increase in Na^+^ influx and, subsequently, water moves into the intracellular environment through osmosis. These processes result in the dehydration of mucous secretions, leading to heightened mucus viscosity and subsequent obstruction, characterized by obstructive tubulopathy and an associated inflammatory response. This physiological manifestation extends to the excretion of sweat and digestive juices [103].

Literature exploring the relationship between CF and sweat highlights the significance of sweat Cl^−^ testing in the diagnosis of this condition. Sweat Cl^−^ concentration serves as a key indicator being diagnostic for CF (Rosenstein and Cutting, 1998). The Gibson and Cooke (1958) method stands as the gold standard for CF diagnosis, involving the iontophoresis-based introduction of pilocarpine into the skin. Sweat collection is achieved through absorption by a filter paper disk or other devices. Originally, 75 mg of sweat was recommended for sampling, with Cl^−^ concentration determination by polarography.

Contemporary methods, such as Macroduct® devices, enable direct sweat collection and transfer to specialized systems for rapid C^−^ measurement using integrated chloridometers. A positive diagnosis of CF is indicated by Cl^−^ values exceeding 60 mmol/L (Rosenstein and Cutting, 1998). A recent innovation in this area is by Emaminejad et al. [104]. The researchers developed a wearable sweat analysis platform with an electrochemically enhanced iontophoresis interface to control the induction of sweat, to overcome the challenge of obtaining a sufficient volume of sweat for on-demand and in-situ analysis. The key innovation of this study was the integration of a miniaturized iontophoresis interface that stimulates sufficient sweat production for robust analysis with the help of an electrical current, overcoming challenges such as electrode corrosion and discomfort for subject [104].

### 5.3 Diabetes

Diabetes is a global public health issue affecting millions of people and its prevalence is growing rapidly. The routine monitoring and treatment of diabetes requires regular assessments of glucose levels. While the conventional method of finger-prick blood collection is fast and satisfactory [105], its inconvenience to patients has prompted the investigation of alternative testing. In response to this demand, there has been a focus on the development of devices for continuous blood glucose monitoring and non-invasive sample analysis [106].

In the pursuit of non-invasive glucose monitoring, wearable devices have shown promise by collecting and analysing perspiration for glucose levels. These sensors can be customisable for multi-substance detection and represent a significant advancement in the area. A study conducted by Moyer et al. [106] utilised a perfusion method for rapid sweat sampling from forearm sites on human subjects. The collected sweat samples were then analyzed for glucose using high-performance liquid chromatography (HPLC) with pulsed amperometric detection (HPAE-PAD) and compared with results obtained from a blood glucose meter. The study included seven individual subjects with diabetes and found a robust correlation between sweat glucose and blood glucose levels. The conclusion drawn was that when sweat is sampled correctly (i.e., preventing contamination from other sources on the skin's surface), it can accurately reflect blood glucose levels. As a result, with correct sample handling and quality controls, sweat has the potential to be reliable medium for precise prediction of blood glucose levels.

A study by Bandodkar et al. [107] developed the first reported temporary tattoo-based, non-invasive glucose monitoring device, combining reverse iontophoresis for glucose extraction with an enzyme-based amperometric biosensor for detection for real-time glucose monitoring. This device extracts glucose from skin interstitial fluid which is then subsequently subjected to evaluation through an enzymatic electrochemical glucose sensor. The fabrication process involved designing sensor patterns in AutoCAD and using stainless steel stencils for fabrication. Ag/Ag Cl^−^ and Prussian blue conductive inks were patterned on the substrate via screen printing, followed by curing at specified temperatures. The working electrode was functionalized with a glucose oxidase solution mixed with chitosan, then dried under ambient conditions [107].

Lee et al. [108] introduced an electrochemical device, incorporating graphene doped with gold, tailored for diabetes monitoring and therapeutic applications. This doping enhances the electrochemical performance of graphene, sufficient to form a wearable patch for sweat-based diabetes monitoring and feedback therapy [108]. Following this Lee et al. [109] developed a wearable, disposable sweat-based glucose monitoring device with integrated feedback transdermal drug delivery. The device uses miniaturized sensors for efficient sweat collection and real-time glucose monitoring, adjusting based on pH, temperature, and humidity. Drugs are delivered through MNs loaded with temperature-responsive nanoparticles, providing controlled, glucose-triggered drug release for diabetes management [109].

Abellán-Llobregat et al. [110] developed two types of highly stretchable, inexpensive, and all-printable glucose sensors using platinum (Pt)-decorated graphite. These sensors include a non-enzymatic and an enzymatic biosensor which utilizes glucose oxidase immobilized on Pt-decorated graphite to measure glucose levels by detecting hydrogen peroxide (H2O2) reduction at −0.35 V via chronoamperometry. The enzymatic sensor demonstrated high sensitivity, selectivity, and a low detection limit in phosphate buffer solution (PBS), with a linear range between 0 mM and 0.9 mM. The sensor was then successfully tested on real human perspiration samples and showed a significant correlation with blood glucose levels [110].

Munje et al. [111] also developed a wearable, flexible electrochemical biosensor using stacked gold/zinc oxide (Au/ZnO) films on porous polyamide for label-free, non-faradaic impedance-based detection of glucose and cortisol in human sweat. Their sensor achieved a low detection limit of 0.1 mg/dL and demonstrated strong correlation with commercial glucose meters, offering dual-analyte monitoring through frequency-specific electrochemical impedance spectroscopy (EIS) measurements.

Notably, recent investigations by Wang et al. [112] developed an ultra-small wearable biosensor system integrating a compact MS02 chip and flexible electrodes for real-time sweat analysis. The miniaturized device accurately monitors multiple biomarkers (glucose, lactate, Na^+^, and K^+^) with performance comparable to commercial electrochemical workstations. Chen et al. [113] have also made substantial progress, introducing innovative methodologies and materials that significantly enhance non-invasive glucose monitoring. The study introduced a non-enzymatic glucose sensor based on surface plasmon resonance (SPR), utilizing a composite film of ZnO nanoparticles and molybdenum diselenide (MoSe_2_) nanosheets. ZnO provided specific glucose binding, while MoSe_2_ enhanced signal amplification due to its high surface area, excellent electron mobility, and biocompatibility. The sensor demonstrated a high sensitivity of 72.17 nm/(mg/mL) and a low LOD of 4.16 μg/mL, with strong selectivity, repeatability, and long-term stability.

### 5.4 AD

AD is a neurodegenerative disorder that not only impacts cognitive function but also extends its influence on various physiological aspects. One important yet underexplored area is the association between AD and sweat composition, particularly sweat Na^+^ concentrations. Sweat Na^+^ plays an important role in thermoregulation, and alterations in its concentration may have implications for the vulnerability of individuals with AD to heat stress.

In a study conducted by Elmstahl and Winge [114], the sweat Na + concentrations in women diagnosed with AD were examined in comparison to healthy volunteers. This investigation aimed to understand the implications for heat stress vulnerability in AD patients. The study cohort comprised 15 women diagnosed with AD and 29 age-matched healthy control women, aged between 76 and 96 years, with a mean age of 85 years. Sweat was induced through pilocarpine iontophoresis under controlled environmental conditions and a significant difference in mean sweat Na^+^ concentration between AD patients and the control group was identified. AD patients exhibited a notably higher mean sweat Na^+^ concentration (91 ± 41 mmol/L, *n* = 11) compared to the control group (62 ± 29 mmol/l, *n* = 27, *P* = 0.0011), signalling altered Na + levels in the sweat of individuals with AD. Interestingly, impaired sweating responses to stimulation were observed in 27% of AD patients, while only 7% of control women displayed a similar response. This impaired sweating in AD patients raises concerns about their susceptibility to heat stress and associated complications.

As a result, further research is warranted to understand more thoroughly the significance of sweat dysfunction in AD. A comprehensive understanding of these mechanisms will not only contribute to the elucidation of the pathophysiology of sweat dysfunction in AD but will also have implications for the management of heat stress in individuals affected by this neurodegenerative disorder.

### 5.5 PD

PD is a neurodegenerative disorder characterized by the progressive loss of dopamine-producing neurons in the brain. Early and accurate diagnosis of PD remains a significant challenge, emphasizing the importance of identifying reliable biomarkers for improved clinical outcomes. Recent investigations have extended to the study of sweat composition as a potential source of diagnostic markers for PD and disease treatment monitoring [115].

In a study led by Sinclair et al. [116], researchers aimed to identify diagnostic markers for PD by examining the regulation of lipid molecular classes in sebum. Employing paper spray ionization coupled with ion mobility mass spectrometry (PS-IMS), the researchers directly analyzed sebum from skin swabs. The study analyzed sebum samples from 150 individuals and found substantial differences in high molecular weight lipids (>600 Da) among PD patients. Accurate mass measurements, tandem mass spectrometry, and collision cross-section measurements were used to obtain putative metabolite annotations for various lipid classes, with a particular focus on triglycerides and larger acyl glycerides. The findings demonstrate the potential of PS-IMS analysis of sebum lipids in identifying PD-specific lipid markers. Notably, the study emphasizes the distinct regulation of triglycerides and larger acyl glycerides in the sebum of individuals with PD. However, further research of these lipid markers in larger cohorts are required to validate their clinical significance and diagnostic potential [116].

The research of George et al. [117] focused on the early detection of PD by investigating non-motor symptoms, specifically speech and smell signatures. The research used MATLAB toolbox and VOC sensors to record and analyse smell of PD and healthy subjects. The findings indicate differences in values computed using VOC sensors for both healthy and PD individuals. Additionally, the study outlines the acquisition process of smell signatures using unscented cotton swabs placed on specific body regions and interfaced with Arduino UNO board. The study proposes a promising approach for PD detection through a combination of speech and smell signatures, offering a non-invasive and reliable technique for early diagnosis [117].

In another study, Sinclair et al. [118] employed dynamic headspace thermal desorption gas chromatography-mass spectrometry (DHS-TD-GC-MS) to directly analyse VOCs from sebum of PD and control subjects. The analysis, involved 100 PD cases (both drug-naïve and medicated) and 29 controls, resulting in an 84.4% accurate classification of PD cases based on the identified volatile compounds. Eight features, including octadecanal, eicosane, and derivatized compound annotations associated with hippuric acid and perillaldehyde, were identified as potential biomarkers of PD due to their downregulation in the control group. This study validates the capability to distinguish between PD and control cohorts by directly measuring volatile compounds from skin swabs collected on the upper back. The findings propose that the VOC profile in sebum could serve as a promising biomarker for PD. The study also acknowledges limitations in identifying specific compounds and suggests future efforts to establish a comprehensive database for improved compound annotation in nonderivatized, biologically relevant VOCs [118].

In a study on continuous sweat monitoring led by Nyein et al. [119], wearable patches were designed to address challenges related to low sweat secretion rates. These patches incorporated microfluidics and hydrophilic fillers to facilitate real-time measurement of sweat secretion rates and integrated electrochemical sensors for pH, chlorine, and levodopa, a drug used in PD treatment. The study includes on-body trials demonstrating the potential of sweat analysis, particularly in monitoring levodopa levels, with implications for personalized medication management in PD patients. The authors highlighted the need for broader population studies to elucidate the relationships between sweat levodopa concentration, plasma levels, and intake dose, while considering diverse factors such as diet, hydration, and physiological conditions [119].

As the literature indicates, ongoing studies in the field are actively working to deepen our understanding of the relationship between PD and sweat composition in order to develop novel diagnostic approaches and therapeutic interventions. The exploration of sweat as matrix of choice for biomarkers discovery contributes to the ongoing efforts to enhance early detection and management strategies for this complex neurodegenerative condition.

### 5.6 Emerging applications

#### 5.6.1 VOCs

VOCs emanating from skin contribute to a person's body odour, and may convey important information about metabolic processes [120]. One of the earliest records of a biomarker study of clinical conditions in sweat is the detection and identification of the trans-3-methyl-2-hexenoic, found in the sweat of schizophrenic patients and conferring it peculiar odour [121]. Additionally, sweat has been investigated as a potential matrix of choice for malaria biomarkers. De Moraes et al. [122] identified 4-hydroxy-4-methylpentan-2-one, nonanal, and toluene as biomarkers in skin volatile samples associated with malaria. The research for biomarkers in sweat also extends to conditions like lung cancer. Calderón-Santiago et al. [72] identified non-anedioic or azelaic acid, monoglyceride MG (22:2), suberic acid, along with a trihexose and a tetrahexose, as potential biomarkers present in human sweat that could be utilised for lung cancer screening. However, the degree to which these compounds can serve as indicators of health and disease remains unclear and continues to an active area of research interest.

In the broader context of VOCs, the literature suggests a growing interest in the use of volatile compounds from sweat as potential indicators for various health conditions, emphasizing the potential of sweat analysis as a non-invasive diagnostic tool. The paper Mitra et al. [123] presents a comprehensive review of the human skin volatolome and demonstrated the presence of a distinct VOC signature or reference profile for healthy skin. The research concludes that the sweat ‘volatolome’ has been shown to consist of aldehydes, carboxylic acids, alkanes, fatty alcohols, ketones, benzenes and derivatives, alkenes and menthane monoterpenoids. The review provides an excellent reference for the ongoing research that continues to explore the volatile signatures of different physiological and pathological states, opening avenues for the development of innovative diagnostic approaches using sweat as a valuable source of biomarkers [123].

#### 5.6.2 Metals analysis in sweat

Metals in the body are fundamental in maintaining a diverse range of biological functions [124]. This raises the question as to whether metal analysis in sweat can convey information on these physiological functions and whether or not they have any health implications, considering that sweat is regarded as means of detoxification or important excretory pathway [125,126].

Research has demonstrated the presence of both essential and toxic metals in sweat. Essential metals like zinc (Zn), copper (Cu), and iron (Fe) are fundamental to various physiological functions, participating in enzymatic activities and redox reactions. Conversely, toxic metals such as lead (Pb), mercury (Hg), cadmium (Cd), and arsenic (As) can accumulate in the body, posing potential health risks. Monitoring the levels of these metals in sweat can provide information into the body's homeostasis and the dynamics of metal bioaccumulation.

For example, Sheng [127] conducted a study involving sweat collection after exercise to evaluate the presence of heavy metals. Samples were digested, and subsequent analysis using atomic absorption spectrophotometry (AAS). The results revealed higher concentrations of Pb, Zn, Cd, Co, Ni and Cu in sweat compared to plasma and urine. Further, Tang [128] employed inductively coupled plasma mass spectrometry (ICP-MS) to quantify trace amounts of heavy metals in exercise-induced sweat. The study demonstrated similar findings to Sheng [127], with elevated concentrations of heavy metals detected in sweat samples. A more recent by Kuan et al. [125] found that the concentrations of heavy metals (Ni, Pb, Cu, and As) in sweat after dynamic exercise were higher than those in sweat after experiencing a thermal sauna environment, indicating that different sweating methods may affect the excretion of heavy metals in sweat.

With regard to review papers Sears et al. [129] and collaborators assessed previous literature on As, Cd, Pb and Hg levels in sweat. The review compiled and analyzed previous studies on the topic and provided additional evidence supporting the presence of these toxic elements in sweat. Sweat has presented higher concentrations of these toxic substances compared to plasma and urine, making it a promising matrix for non-invasive monitoring and evaluation of bioaccumulated heavy metals.

## 6 Key challenges and future perspectives

### 6.1 Practical considerations

Sweat is well suited as a matrix for wearable sensor technologies due to its simple extraction and, as discussed, is a source of various biomarkers Combined with the portability, non-invasive and ease/comfort afforded by wearable sensor technologies, present an attractive approach to individualised health monitoring However, whilst the body naturally and continually secretes sweat both during sedentary and routine activities the low secretion rates and evaporation continue to present as key challenges. As such, issues such as sweat collection, sample degradation, individual variations, detection method, and sensitivity, as well as data analysis and interpretation, will collectively shape the overall commercial viability outcomes of any proposed sweat sensors.

To overcome such challenges, one avenue of research looks at leveraging microfluidic channels to improve the performance and sensitivities of the sensor by manipulating and controlling the flow of very small amounts of fluids within channels or structures typically at the microscale. Goals of the microfluidic design or architecture are to direct the flow of sweat into the device, as well as to avoid oversaturation of both the channels and sensors. This can be accomplished using combinations of super hydrophilic and super hydrophobic regions, utilizing manual pressure or pull-tab pumps and integration of valves either controlled electronically or based on exploiting the natural pressure build-up that occurs by sweat glands [51].

For example, Nyein et al. [119] developed a wearable patch that utilised a hydrophilic filler to absorb and channel sweat from the skin into a microfluidic system for analysis and utilised electrochemical sensors for pH, chlorine, and levodopa monitoring, a precursor to dopamine. This combination of electrochemical sensors creates new opportunities to study how the body's endogenous sweating response relates to stress, metabolic conditions, and potential neurological afflictions, amongst other applications of physiology [119]. Further, the use of fluid control techniques like iontophoresis which leverages electrical forces to control the movement of charged particles (like ions) within a fluid, avoiding the need for physical valves, can simplify the design and structure of the fluid control system. The use of iontophoresis for electrically inducing migration of ions/molecules across the skin surface can be used to both deliver and extract (reverse iontophoresis) desired compounds and can enable controlled, on-demand localized sampling [51].

For example Kim et al. [130] developed a flexible wearable device (similar to that of a printed tattoo) that simultaneously collects interstitial fluid (ISF) through reverse iontophoresis and induces sweat using a drug (pilocarpine). The device then utilizes electrochemical biosensors to analyse ISF glucose and sweat alcohol in real-time from two epidermal biofluids at the same time, in two different physical locations [130].

Advancements in electrochemical sensors are also occurring. Biochemical sensors typically employ optical methods (fluorescence and colorimetry) and electrochemical sensing which base their measurements on amperometry, potentiometry, and voltammetry. The various sensing modalities offer various advantages, however they can also present specific challenges and careful consideration is required, depending on the intended application and vary in the selectivity and sensitivity [131]. For example, potentiometry, which is an electrochemical technique, is based on the measurement of the potential of an electrode system and offers analytical advantages such as quick response times, broad linear working ranges, high sensitivity, and precise and selective determination of various ionic species, along with physical advantages such as low energy consumption, affordability and straightforward preparation [132]. They are also adaptable, along with other electrochemical based sensors for printing technologies which support miniaturization [133]. Nevertheless, the accuracy of electrochemical biosensors can be influenced by variations in pH and electrolyte concentration in the sweat due to interfering background signals or enzyme activity. However, Ibanez-Redin et al. [134] were able to address this challenge using screen-printed electrode arrays where ink is applied using a design stencil to a substrate resulting in multiple electrodes for a more comprehensive and efficient sensing platform. In doing so, the effect from such interferences could be determined in order to optimize the device for the sensitive and accurate detection of urea in sweat.

Another study by Vernekar et al. [135] demonstrated improved sensitivity and stability for electrochemical detection of mefenamic acid (MA) through a novel carbon paste electrode (CPE) modified with glucose and cetyltrimethylammonium bromide (CTAB). This dual modification enhanced the interaction between the analyte and electrode surface, making the system more effective for detection in complex samples. As a result, the CPE was able to achieve the ultrasensitive detection of MA, with a linear dynamic range of 2.5 × 10^−8^ to 5.0 × 10^−4^ M and a detection limit of 1.01 (±0.03) nM. The sensor showed negligible interference from co-existing molecules and was successfully applied to real sample analysis, with recovery rates of 92.70%–99.16% in urine and 96.0%–98.5% in pharmaceutical tablets.

However, one of the earliest and influential demonstrations of fully integrated wearable sweat sensors was by Gao et al. [136] who developed a flexible, skin-conforming platform capable of multiplexed detection of sweat metabolites (e.g., glucose, lactate) and electrolytes (e.g., Na^+^, K^+^), with on-board signal conditioning and wireless data transmission. It utilised an integrated sensing array for simultaneous and selective screening of a panel of biomarkers in sweat. This study demonstrate the feasibility of an integrated multiplexed sensing system and and presents as a benchmark for integration and functionality in wearable health monitoring.

### 6.2 Clinical translation barriers

While innovation in wearable sweat technology continues to advance, the successful translation and routine clinical application of point-of-care sweat sensors remains a significant challenge. This includes the ability to predict pathologies before traditional symptoms appear, the identification and validation of clinically relevant biomarkers, and the subsequent design of sensors capable of detecting these validated biomarkers at trace and highly variable levels.

#### 6.2.1 Data interpretation and predictive analytics

The ability to predict pathologies before they appear is an important challenge to overcome, as it would enable earlier intervention, improve patient outcomes, and shift healthcare toward a more preventative model. One approach to address this challenge is to combining wearable sensors with data mining [137]. In order to be able to predict pathologies before symptom manifestation, real-time and continuous human physiological assessment requires normalization of the analyte concentrations to adequately assess normal day-to-day various within an individual. Wearables that continuously monitor such physiological events also need to consider events that impact human performance, such as dehydration, fatigue, and disease with the ability to differentiate between such events [138]. Currently, limitations of sensor technology include noise and drift, with fluctuating data or differentiating normal spikes or legitimate signals from abnormal or erroneous events. Further limitations arise from extracting useful information from large amounts of data and identifying meaningful trends within the data for easier interpretation by the consumer to access clear and actionable insights, allowing them to quickly understand their health status [139].

However, AI combined with the internet of things (IoT) opens the possibility for a strong tool to better handle such large data sets and to enable for greater accuracy in predictions and extraction of meaningful insights [139]. For example, Wang et al. [140] presented the first study on predicting hydration status using machine learning models. During exercise, four noninvasive physiological and sweat biomarkers (heart rate, core temperature, sweat Na + concentration, and whole-body sweat rate) were measured. Three machine learning models were then applied to predict percentage body weight loss as an indicator of dehydration, and their prediction accuracies were compared. The results, based on a single-subject study, showed similar mean absolute errors across models, with nonlinear models generally outperforming the linear model [140].

Following on, a study reported in 2025 by Chenani et al. [141] present a stretchable, adhesive bilayer hydrogel-based patch designed for continuous monitoring of sweat pH and glucose levels, coupled with AI-assisted smartphones. The study utilised machine learning models, including random forest (RF) and convolutional neural networks (CNN), to analyse sweat data. The models achieved a high degree of accuracy, with a coefficient of determination (*R*^2^) of ∼0.99 for pH (3–9) and glucose concentrations up to 0.5 mM, validating the patch's reliability against standard methods like HPLC. Such studies marks a significant step toward the use of machine learning for personalized health monitoring using noninvasive wearable sensor technologies.

The use of big data can also be used to uncover correlations between sensor readings and health status, even without a complete understanding of their causal relationship. The utilization of statistical methods, pattern recognition, and AI to extract correlations to enable health status prediction will be critical to utilizing wearable sensors for human health monitoring. However, the integration of AI in wearable biosensors also extends to other considerations. These include the high-power computation and data storage capacity in order to store and process information and communicate between cloud servers from personal devices, as well the reliability of adaptive learning and synthetic data [142]. Other issues that would need to be considered include the associated privacy concerns in handling and protecting people's data [142].

#### 6.2.2 Identifying clinically relevant biomarkers

One of the most critical challenges before developing sensors for clinical use and point-of-care health monitoring is identifying clinically significant biomarkers and translating them into measurable analytes in sweat. This involves understanding the molecular signature of the analyte across various biological fluids, including blood, ISF, oral fluid, and sweat [143].

Biomarkers serve as biological indicators that span across several separate categories depending on their role. For example prognostic biomarkers, which are objectively measured biological characteristics associated with a particular clinical outcome or disease progression of a particular patient; diagnostic biomarkers which are measurable biological molecules that indicate the presence of absence of a disease or conditions and; predictive biomarkers which can indicate whether a patient is likely to respond to a particular treatment or therapy [144]. Other categories include biomarkers related to susceptibility which provide an indication of an individual's susceptibility to disease, monitoring biomarkers which can track disease progression or treatment effects, response biomarkers which verify biological responses after treatment or exposure, guiding optimal dosage and safety biomarkers which detect potential toxic responses to therapies or environmental exposures [143]. However, their discovery and validation processes face hindrances such as the ability to capture all analytes, better methods for targeting polar metabolites and a lack of standardized protocols for analytical studies, as well as extending considerations to sample collection, handling, and storage. Identifying the origin of the metabolites present in sweat is also a challenge [143].

In contrast to outcome-driven biomarkers, which consider individual analytes, another approach to biomarker discovery is to consider the biological system as a whole, otherwise known as systems biology. System biology driven biomarkers results from looking at biological systems from a holistic standpoint (as opposed to each individual part) using omics data, computational modelling and network analysis in order to provide a more comprehensive understanding of biological interactions that are occurring [144]. Such an approach is supported by the current improvements in advanced scientific methods such as proteomics and metabolomics, which continue to deliver valuable information about important substances in the body. However, more research is required to establish the clinical significance of the metabolome-proteome in biomarker discovery and system biology. Key limitations include a lack of studies correlating altered sweat pathways with specific conditions and insufficient longitudinal data to monitor disease progression or treatment effects [145].

#### 6.2.3 Challenges in forensic and clinical applications

In forensic and clinical toxicology, sweat has continued to be explored as a matrix of choice for drug testing. However, low sample volumes, sweat composition variability, contamination risks and sensitivity and specificity of detection continue to remain as key challenges. Over the years, studies have demonstrated that certain drugs can be detected in sweat specimens, as outlined earlier. The emergence of NPS opens a new line of investigation with the possibility of analysing these substances in sweat specimens. Sample collection and preparation have been proven to be easier and simpler for sweat samples compared to other biological matrices. Nevertheless, to achieve this goal, analytical research on the development and validation of methods continues to be required. Furthermore, as some of these drugs are highly potent and typically found in the sub ng/mL range in blood, highly sensitive techniques will be required for untargeted or targeted analysis of NPS in sweat.

The study by Jagannath et al. [5] also demonstrates important progress in the field of sweat analysis, in the context of clinical applications, by detecting inflammatory cytokines in passively expressed sweat from human subjects using a wearable sensor. The study showed the ability to distinguish between healthy individuals and those with infections such as fever or flu. This highlights the potential for sweat-based sensing to go beyond commonly targeted electrolytes and metabolites. Cytokines are not yet widely detected as analytes in wearable sweat sensors, and this work is an important step forward for future applications in immune monitoring and broader health surveillance. In a more recent study Chu et al. [146], then created a flexible, wearable electrochemical fabric for IL-6 monitoring. This fabric used an aptamer-functionalized carbon nanotube/graphene composite fibre that demonstrated satisfactory sensing capability of *in vitro* and *in situ* L-6 determination. The electrochemical fabric can track other biomarkers by replacing the coupling aptamer, suggesting the potential for the monitoring fabric to serve as a universal platform for sweat analysis and personalized health monitoring [146]. This is supported by findings of earlier work by Marques-Deak et al. [147] which indicate that cytokine levels measured in sweat are informative of circulating levels. The study used sweat patches combined with recycling immunoaffinity chromatography (RIC) coupled with laser-induced fluorescence (LIF) detection. RIC-LIF is a highly sensitive and specific detection method and enables the detection of a much larger number of cytokines per unit of fluid compared to other techniques [147].

### 6.3 Sweat sensor design, functionality and other considerations

Another challenge for wearable sweat biosensors for real-time continuous monitoring is comfort, as well as reducing the mismatch that can occur in the physical properties at the electronic-skin interface [148]. A recent review by Dkhar et al. [149] presents the significant advancements occurring in wearable biosensors, particularly those based on skin-like and textile materials and the role of wearables in detecting diseases like coronavirus disease 2019 (COVID-19) and identifies ongoing challenges in improving sensor accuracy and applicability for personalized therapy. Other important considerations also include issues such as long-term monitoring and how this relates to the mechanism for collection and analysis of sweat, as well as the supply and management of energy to the device, and finally the establishment of the relationship between monitoring data and human health need to be overcome in order to develop a commercialized wearable sweat sensor [150].

## 7 Concluding remarks

The use of sweat as an alternative specimen for biological analyses is well recognised as a non-invasive sampling technique with broad applicability in the field of health monitoring. Current research supports its suitability as a matrix for analysing biomarkers that are indicative of health status for a range of pathologies, as well as human performance monitoring. With the demand for non-invasive testing technologies that offer convenience, user-friendly interfaces, portability, and wireless connectivity, sweat as the biological matrix for analysis is anticipated to continue to receive attention for biomarker identification for health monitoring. This will be supported by the developments occurring in advanced analytical techniques that can provide comprehensive data sets with increased selectivity and sensitivity. However, the key challenge is moving from labour-intensive manual, endpoint-analyses, into a wearable, continuous monitoring system. This system requires interfacing of microfluidics, sensing, and computation within an electronic platform that is attached to a region of the body and that offers wireless connectivity to a personal device, from which an app will automatically be able to transfer data into meaningful information for the wearer. It is anticipated that advances in the performance and miniaturization of integrated circuits, stretchable electronics, wireless connectivity, and longer battery life, will confer improvement in analyte detection through improving the interface between the device and the source, and increasing the sensitively, along with improved wearability, battery life and predictive AI. The integration of these key technologies will enable a greater understanding of the composition of composition and its changing dynamics and its relationship with health, presenting opportunities to play a key role in the future of personalized healthcare and disease management through a non-invasive, easier to use consumer technology. The challenges and opportunities discussed herein support the need for continued innovation and collaboration.

## CRediT authorship contribution statement

**Dayanne Mozaner Bordin:** Writing – review & editing, Writing – original draft, Visualization, Conceptualization. **Janice Irene McCauley:** Writing – review & editing, Writing – original draft. **Eduardo Geraldo de Campos:** Writing – review & editing, Writing – original draft, Conceptualization. **David Bishop:** Writing – original draft, Supervision. **Bruno Spinosa De Martinis:** Writing – original draft, Supervision.

## Declaration of competing interest

The authors declare that they have no known competing financial interests or personal relationships that could have appeared to influence the work reported in this paper.
