# Supplementary material for: Sweat as a diagnostic biofluid: analytical advances and future directions
Source: J Pharm Anal. 2025 Oct 21;16(7):101473. doi: 10.1016/j.jpha.2025.101473 (PMC13400351; doi:10.1016/j.jpha.2025.101473)
Supplement: Multimedia component 1 [file mmc1.pdf]

Dayanne Mozaner Bordin

From: Janice McCauley
Sent: Thursday, 24 July 2025 5:27 AM
To: Dayanne Mozaner Bordin
Subject: Fw: Copyright Permission to Reproduce Schematic Image of the DrugWipe Screening Test Device

---

From: Verena Zimmermann <v.zimmermann@securetec.net>
Sent: Wednesday, 23 July 2025 8:50 PM
To: Janice McCauley <Janice.McCauley@uts.edu.au>
Cc: Stephen Kelly <Stephen.Kelly@pathtech.com.au>
Subject: [EXTERNAL] WG: Copyright Permission to Reproduce Schematic Image of the DrugWipe Screening Test Device

This message needs your attention

- This is their first email to your company.

Mark Safe Report

Want to know more? thinksecurely.uts.edu.au

Dear Dr McCauley,

We hereby grant you the permission to reproduce and adapt the image of our DrugWipe A test.

We would be grateful if you could provide us with the draft publication of your paper "Sweat as a Diagnostic Biofluid: Analytical Advances and Future Directions" before it will be published.
Thank you very much.

Best regards
Verena Zimmermann

Verena Zimmermann
CEO

Tel: +49 89 203080-1650
Fax: +49 89 203080-1652
Email: v.zimmermann@securetec.net
www.securetec.net

SECURETEC
DETEKTIONS-SYSTEME AG

Securetec Detektions-Systeme AG
Lilienthalstraße 7 · 85579 Neubiberg · Deutschland
Vorstand/Board of directors: Verena Zimmermann, Stefan Masur
Handelsregister/Commercial Register: HRB 136885 (RG München)

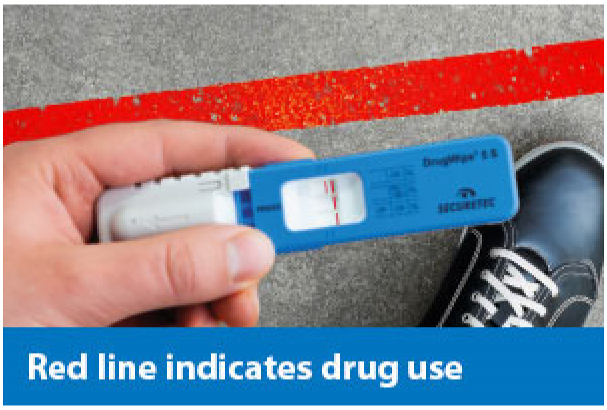

Von: Janice McCauley <Janice.McCauley@uts.edu.au>
Gesendet: Freitag, 18. Juli 2025 06:25
An: Stephanie Konopac <Konopac@securetec.net>
Betreff: Copyright Permission to Reproduce Schematic Image of the DrugWipe Screening Test Device

Dear Customer Support,

I am writing to request permission to reproduce and adapt, as a schematic, the image of the DrugWipte Screening Test Device featured on your website:

https://www.securetec.net/en/products/drug-screening-test/

Drug screening test DrugWipe® A - Securetec Detektions-Systeme AG

Accurate detection of drug use Application: Customs, border control, occupational safety Results available in: 3-5 minutes Fast detection of the 5 most common drug groups cannabis, opiates, cocaine, amphetamines and methamphetamines (including MDMA, ecstasy) Features of the drug wipe test The DrugWipe ® A test reliably detects illegal drugs on surfaces, skin and saliva. As a combined ...

www.securetec.net

I would like to include this schematic representation in a review paper titled "Sweat as a Diagnostic Biofluid: Analytical Advances and Future Directions", which is being prepared for submission to the peer-reviewed journal The Journal of Pharmaceutical Analysis (Impact Factor: 14.026). The image would be used solely for non-commercial, academic purposes, with full credit given to Securetec.

Please let me know if you are able to grant permission, and if so, whether there are any specific conditions or attribution guidelines you would like me to follow.

Thank you for your time and consideration.

Kind regards,

Dr Janice McCauley
Lecturer - Analytical Chemistry
School of Mathematical and Physical Sciences, University of Technology Sydney
M. +61 403 138 254
PO Box 123. Broadway NSW 2007 Australia

uts.edu.au

UTS CRICOS Provider Code: 00099F DISCLAIMER: This email message and any accompanying attachments may contain confidential information. If you are not the intended recipient, do not read, use, disseminate, distribute or copy this message or attachments. If you have received this message in error, please notify the sender immediately and delete this message. Any views expressed in this message are those of the individual sender, except where the sender expressly, and with authority, states them to be the views of the University of Technology Sydney. Before opening any attachments, please check them for viruses and defects. Think. Green. Do. Please consider the environment before printing this email.
